# Identification of sex-linked SNP markers using RAD sequencing suggests ZW/ZZ sex determination in *Pistacia vera* L.

**DOI:** 10.1186/s12864-015-1326-6

**Published:** 2015-02-18

**Authors:** Salih Kafkas, Mortaza Khodaeiaminjan, Murat Güney, Ebru Kafkas

**Affiliations:** Department of Horticulture, Faculty of Agriculture, University of Çukurova, Adana, Turkey

**Keywords:** Pistachio, *Pistacia vera*, Sex determination, RADseq, SNP, SNaPshot, HRM

## Abstract

**Background:**

Pistachio (*Pistacia vera* L.) is a dioecious species that has a long juvenility period. Therefore, development of marker-assisted selection (MAS) techniques would greatly facilitate pistachio cultivar-breeding programs. The sex determination mechanism is presently unknown in pistachio. The generation of sex-linked markers is likely to reduce time, labor, and costs associated with breeding programs, and will help to clarify the sex determination system in pistachio.

**Results:**

Restriction site-associated DNA (RAD) markers were used to identify sex-linked markers and to elucidate the sex determination system in pistachio. Eight male and eight female F_1_ progenies from a *Pistacia vera* L. Siirt × Bağyolu cross, along with the parents, were subjected to RAD sequencing in two lanes of a Hi-Seq 2000 sequencing platform. This generated 449 million reads, comprising approximately 37.7 Gb of sequences. There were 33,757 polymorphic single nucleotide polymorphism (SNP) loci between the parents. Thirty-eight of these, from 28 RAD reads, were detected as putative sex-associated loci in pistachio. Validation was performed by SNaPshot analysis in 42 mature F_1_ progenies and in 124 cultivars and genotypes in a germplasm collection. Eight loci could distinguish sex with 100% accuracy in pistachio. To ascertain cost-effective application of markers in a breeding program, high-resolution melting (HRM) analysis was performed; four markers were found to perfectly separate sexes in pistachio. Because of the female heterogamety in all candidate SNP loci, we report for the first time that pistachio has a ZZ/ZW sex determination system. As the reported female-to-male segregation ratio is 1:1 in all known segregating populations and there is no previous report of super-female genotypes or female heteromorphic chromosomes in pistachio, it appears that the WW genotype is not viable.

**Conclusion:**

Sex-linked SNP markers were identified and validated in a large germplasm and proved their suitability for MAS in pistachio. HRM analysis successfully validated the sex-linked markers for MAS. For the first time in dioecious pistachio, a female heterogamety ZW/ZZ sex determination system is suggested.

## Background

The *Pistacia* genus is a member of the Anacardiaceae family and consists of at least 11 species [1,2]. *Pistacia vera* is the only commercially important species, and is believed to be the most ancestral species, other species are probably its derivatives [3]. In the wild, the species grows as a forest tree; some wild species are used as rootstock for *P. vera* [4]. With a few exceptions the sex habit is dioecious, with trees bearing wind-pollinated apetalous flowers [5]. Iran, USA, Turkey, and Syria are the main pistachio producers in the world, contributing over 90% of production [6]. Pistachio has a chromosome count of n = 15 [7] and according to flow cytometry analysis a haploid genome size of approximately 660 Mbp [8].

In recent decades, numerous DNA-based molecular marker systems have been developed for marker-assisted selection (MAS) in breeding programs. Next-generation sequencing (NGS) technology provides an effective tool for MAS through generating huge number of DNA markers within a short period. Therefore, NGS-based marker systems allow highly efficient marker development for MAS in plant breeding. One such system is restriction site-associated DNA sequencing (RADseq), which detects polymorphic variants neighboring particular restriction enzyme recognition sites [9]. RADseq has been used to detect single nucleotide polymorphism (SNP) in a variety of plant species, with or without an available reference genome [10,11]. RADseq was applied in plants for marker development linked to anthracnose and stem blight diseases in lupin (*Lupinus angustifolius* L.) [12,13], and for SNP discovery and genetic mapping in *Lolium perenne* L. [14,15], eggplant (*Solanum melongena* L.) [10], grape (*Vitis vinifera* L.) [16], globe artichoke (*Cynara cardunculus* L.) [17], rapeseed (*Brassica napus* L.) [18], and sunflower (*Helianthus annuus* L.) [19]. RADseq has also been used in aquaculture to find sex markers in *Hippoglossus hippoglossus* [20] and *Lepeophtheirus salmonis* [21].

Approximately six percent of angiosperm species are dioecious [22]. Dioecy may have evolved from hermaphrodite or monoecious ancestors by two independent mutations: a first mutation causes male sterility, whereas a second mutation results in decreased female fertility, leading to functional dioecy [23]. Sex determination mechanisms in plants are diverse, and may involve sex chromosomes, such as in *Silene* [24], *Carica* [25], and *Actinidia* [26] or individual sex gene(s), e.g., *Mercurialis* [27] and *Cucumis* [28]. In *Pistacia*, the genetic mechanism of sex determination remains unknown.

Sex chromosomes only evolve in dioecious species when two sex-determining genes are closely linked on the same chromosome with complementary dominance [29]. An essential event in sex chromosome evolution is the suppression of recombination between the two sex determination genes. The first step in sex chromosome evolution is the entity of male and female sterile mutations, leading to the development of unisexual reproductive structures [30]. Flower primordia in the buds of female and male pistachios are similarly initiated in trees of both sexes, with differences between the two sexes becoming apparent early in development; in both cases, the development of organs of opposite sex becomes arrested at the primordial stage [31].

As *Pistacia* has a long juvenility period, sometimes greater than 10 years, MAS may greatly facilitate pistachio cultivar-breeding programs. Early diagnosis of seedling sex would greatly assist cultivar-breeding programs, nursery management, and germplasm collection.

A Randomly Amplified Polymorphic DNA (RAPD) marker was reported to distinguish male and female *P. vera* seedlings [32]. Bulk segregant analysis screening identified a single sex marker (OPO08_945_). However, this RAPD marker was ineffective for sex determination in other *Pistacia* species [33]. Yakubov *et al.* [34] converted the RAPD marker into a Sequenced Characterized Amplified Region (SCAR) marker; however, amplification of the SCAR marker was observed in both sexes. The authors sequenced the bands amplified in the male and female, revealing 909-bp and 905-bp length fragments, respectively. These presented high homology (95%), and contained several point mutations. The authors designed new SCAR primers based on the polymorphic locus, these amplified a 297-bp DNA fragment in both sexes. The SCAR primers were tested in six female and six male *P. vera* individuals. The authors indicated that a combination of touchdown PCR program and SCAR primers enabled the development of a female-specific marker in *P. vera*. Recently, Esfandiyari *et al.* [35] tested the SCAR primer pair in four *P. atlantica* Desf. and four *P. khinjuk* Stock individuals; they reported that the SCAR marker designed by Yakubov et al. [34] could effectively distinguish sex in wild species. However, testing of the RAPD and SCAR primers in a *P. vera* (Siirt × Bağyolu) F_1_ segregating population, and in a large germplasm collection in our laboratory, revealed false negatives in some female individuals and false positives in some male individuals, including the Bağyolu male parent. Our analysis also demonstrated that the SCAR marker could not distinguish sex in wild *Pistacia* species. Therefore, a new strategy is necessary to find sex-linked markers and to elucidate the sex determination mechanism in pistachio.

This study aimed to identify SNP loci linked to sex in pistachio through NGS-based RADseq, using mature female and male individuals in a Siirt × Bağyolu F_1_ segregating population. Sex-linked SNP markers were validated both in segregating populations and in a large pistachio germplasm by SNaPshot analysis. Their application was then tested by high-resolution melting (HRM) analysis for cost-effective marker-assisted selection in cultivar-breeding programs. Inheritance of the markers suggested a sex determination system for dioecious pistachio. There is currently a lack of adequate information on the pistachio genome in the literature. This study is the first to report use of NGS, SNaPshot and HRM for SNP discovery and application in pistachio.

## Results

### SNP discovery in pistachio by RADseq

Restriction site-associated DNA sequencing (RADseq) of 9 male individuals and 9 female individuals in a *Pistacia vera* L. Siirt × Bağyolu F1 population (including the two parents) was performed in two lanes of a Hi-Seq 2000 sequencing platform. In all, 450,721,882 reads, comprising approximately 36,959,194,324 nucleotide sequences were obtained following cleaning of multiplex identifier (MID) sequences. 18.49 Gb of data was obtained from female plants and 18.47 Gb from male plants. The average data comprised 2.05 Gb per plant, with variation between plants from 0.83 to 3.26 Gb (Table 1). Average coverage depth per read tag among individuals varied from 23.9× to 63.8× with an average of 47.3×. Considering that the genome size of pistachio is ~660 Mbp, sequencing coverage of the 18 individuals varied from 1.3× to 4.9×, with an average of 3.1×. The total number of RAD tags for each individual plant varied between 410,036 and 657,285 with an average of 519,937.

**Table 1:** **RADseq results in 18 plants using Hi-seq 2000 NGS platform**

**Parents and F1 plants**	**Read number**	Total RAD sequence (bp)*	**Number of RAD tags**	Total sequence of RAD taqs (bp)**	Coverage depth***	Sequence coverage****	**GC rate (%)**	**Total SNP**	**Heterozygous SNP rate (%)**
Females									
Siirt	15,873,498	1,301,626,836	422,422	34,638,604	37.6	2.0	37.65	90,999	17.3
P-F-1	24,754,856	2,029,898,192	442,751	36,305,582	55.9	3.1	37.12	98,348	26.9
P-F-2	22,527,994	1,847,295,508	513,815	42,132,830	43.8	2.8	37.60	97,562	28.0
P-F-3	28,035,806	2,298,936,092	513,654	42,119,628	54.6	3.5	37.07	99,776	25.3
P-F-4	33,578,112	2,753,405,184	560,479	45,959,278	59.9	4.2	36.86	101,339	30.5
P-F-5	23,563,272	1,932,188,304	539,390	44,229,980	43.7	2.9	37.47	98,767	31.9
P-F-6	23,374,790	1,916,732,780	523,591	42,934,462	44.6	2.9	37.38	98,876	29.9
P-F-7	28,326,906	2,322,806,292	539,702	44,255,564	52.5	3.5	36.88	100,355	27.4
P-F-8	25,474,290	2,088,891,780	431,895	35,415,390	59.0	3.2	37.64	96,551	17.5
Subtotal	225,509,524	18,491,780,968	4,487,699	367,991,318	451.6	28.0	-	882,573	-
Subaverage	25,056,614	2,054,642,330	498,633	40,887,924	50.2	3.1	37.30	98,064	26.1
Males									
Bağyolu	27,187,532	2,229,377,624	552,533	45,307,706	49.2	3.4	38.00	79,746	33.1
P-M-1	22,615,328	1,854,456,896	498,044	40,839,608	45.4	2.8	36.98	98,046	27.2
P-M-2	10,108,252	828,876,664	422,176	34,618,432	23.9	1.3	37.10	75,521	20.5
P-M-3	19,417,040	1,592,197,280	541,272	44,384,304	35.9	2.4	37.48	95,941	26.7
P-M-4	11,079,312	908,503,584	410,036	33,622,952	27.0	1.4	38.77	72,909	18.6
P-M-5	38,882,284	3,188,347,288	657,285	53,897,370	59.2	4.8	37.65	102,340	30.5
P-M-6	26,158,302	2,144,980,764	569,710	46,716,220	45.9	3.2	37.58	100,049	27.0
P-M-7	29,947,304	2,455,678,928	596,268	48,893,976	50.2	3.7	37.51	99,531	27.5
P-M-8	39,817,004	3,264,994,328	623,841	51,154,962	63.8	4.9	37.41	103,115	27.8
Subtotal	225,212,358	18,467,413,356	4,871,165	399,435,530	400.6	28.0	-	827,198	-
Subaverage	25,023,595	2,051,934,817	541,241	44,381,726	44.5	3.1	37.61	91,911	26.5
Total	450,721,882	36,959,194,324	9,358,864	767,426,848	852.2	56.0		1,709,771	
Average	25,040,105	2,053,288,574	519,937	42,634,825	47.3	3.1	37.45	94,987	26.3

*Total RAD sequence was calculated by multiplying read number by 82 bp read length of Illumina.

**Total sequence of RAD taqs was calculated by multiplying number of RAD tags by Illumina 82 bp read length.

***Coverage depth was calculated by dividing Illumina read number by number of RAD taqs.

****Sequence coverage was calculated by dividing total RAD sequence (bp) by the estimated genome size (660 Mbp) of pistachio.

The number of single nucleotide polymorphisms (SNPs) for each individual varied from 72,909 to 103,115 with an average of 94,987. The average rate of heterozygote SNPs was 26.3%. On average, there was one SNP for each 449 bp in pistachio. SNPs were detected more frequently in female individuals (one SNP per 418 bp) than in male individuals (one SNP per 483 bp). Similarly, SNPs were observed more frequently in the female parent cv Siirt (one SNP per 381 bp) than in the male parent cv Bağyolu (one SNP per 568 bp).

### Identifying SNP markers linked to sex in pistachio

Following removal of monomorphic RAD reads between the two parental plants of the Siirt and Bağyolu cultivars, 33,757 polymorphic SNP markers were detected across 18 individuals. These markers were subjected to sex marker identification, and 38 putative sex-associated SNP loci were determined in 28 RAD reads. Among these, suitable primer designs were possible for 13 SNP loci from 11 RAD reads (Table 2). The others did not have enough sequence for primer design in one of two ends of a 82 bp Illumina read. Two reads (SNP-PIS-133396 and SNP-PIS-135862) had two adjacent SNP loci; therefore 11 reads were used for the primer design.

**Table 2:** **Marker name, position and sequences of candidate SNP sex markers in pistachio**

**No**	**Marker name**	**SNPs**	**Positions**	**Sequence (5′-3′)**
1	SNP-PIS-1319	Y-Y	28-77	AATTCGTATAGCCCGTGAGAATACATG(Y)GGATGAGGGCTATACATGAGAGAGAGTACACTCACATGGCCAAGGGTT(Y)CGAAT
2	SNP-PIS-29689	Y-R	33-71	AATTCAACATCTTATAAAGCGAAATCACTTCA(Y)AATAATGCTTCTTCTTTGCAAGTGCACCAAACAATAT(R)TTGAATGATGA
3	SNP-PIS-112277	Y-R	10-61	AATTCGTTA(Y)CTAGAGGGTGATTTTAAAACTCTTACAGACACAAAACCATGACAATAATT(R)AAGGAAGAAAATTCAGCATGC
4	SNP-PIS-120693	M-W	19-57	AATTCAATGATCTAGATT(M)AAAGAAGGCATTGGATGTTGTGTATTGTCATTTGTAA(W)AATATCTTGGTGTGTAAAATGTGTA
5	SNP-PIS-127343	S	27	AATTCACCAATATTTTACTGCAATAA(S)TAAGAATGTAATGACAGGGTGAGTGAAAATGGTAGATTAAAATTTTAAGGAAATG
6	SNP-PIS-133396	S-Y	46-47	AATTCTCCTCTGTTTTTTGGGCAAACCGCAAAGAAGATTAAAGTA(S)(Y)TGATCCATGATCTTCAAGTTTCAGTACTATTCATA
7	SNP-PIS-135862	Y-Y	40-41	AATTCTTTGGTTTTGTGTCTGAATGTGGATAATATATGG(Y)(Y)GCCTCATGTTGATTATGGGAAATGTGCATGGAAATAGTATC
8	SNP-PIS-136404	K	23	AATTCTTTTAGGGGTTGTCAAA(K)TGACCGGATTCCTCACAAATTCAATTGCCAACTCTAAAGCTGGCAAGAAATTCTTTAGC
9	SNP-PIS-167992	W	36	AATTCAAACGAAAAATAACTTCATAGCGTGAGCTC(W)TTGTTCCACCTSTAACCGCAACCCTAAGCTGCAATTGATCACTTCC
10	SNP-PIS-174431	M	56	AATTCCATTACTTCAACAAGTCTCTAGCCGCGTACATATAAAAATTAACTACTCA(M)AGTGAAAGTGGAYAAATTGTTAAGTT
11	SNP-PIS-176863	R	57	AATTCCACATTTGACMAGGGTTGGAACTTTTGAGGTGGATGTGAGCTTGGAAGGTA(R)TATCATACTTTGCAGACAGGTCGAT

The SNaPshot is a minisequencing and primer extension–based method developed for the analysis of SNPs. Therefore, it was used for validation of sex-associated SNP markers in this study. SNaPshot analysis was initially performed in 42 F_1_ mature progenies with known sex. In the next step, if a SNaPshot primer set could perfectly distinguish sex in pistachio, the primer set was further tested in 17 male and 47 female cultivars or genotypes of diverse origin in the germplasm collection, as well as in 60 open-pollinated mature progenies derived from Siirt and Ohadi cultivars (15 males and 15 females from each cultivar). If the SNP locus distinguished sex correctly in all 166 individuals, then it was considered a valid sex-linked locus in pistachio. Next, forward and reverse SNP flanking primers were used for high resolution melting (HRM) analysis by real-time PCR. Since HRM analysis is a rapid and a cost-effective assay for marker-assisted selection (MAS) in the breeding programs. Sequences of the SNP flanking primers and single-base extension primers used are given in Table 3.

**Table 3:** **Primer sequences of candidate SNP sex markers used in for SNaPshot and HRM analysis in pistachio**

**No**	**Marker name**	**Primer**	**Position**	**Sequence (5′-3′)**	**Product size (bp)**
1	SNP-PIS-112277	Forward		TTACAGACACAAAACCATGACAA	50
		Reverse		GCATGCTGAATTTTCTTCCT	
		Single base extension	61-R	GCATGCTGAATTTTCTTCCTT	
2	SNP-PIS-127343	Forward		TCACCAATATTTTACTGCAA	56
		Reverse		CCATTTTCACTCACCCTGTC	
		Single base extension	27-S	CACCCTGTCATTACATTCTTA	
3	SNP-PIS-133396	Forward		GCAAACCGCAAAGAAGATTA	52
		Reverse		ACTGAAACTTGAAGATCATGGA	
		Single base extension	46-S	CAAACCGCAAAGAAGATTAAAGTA	
			47-Y	GTACTGAAACTTGAAGATCATGGATCA	
4	SNP-PIS-135862	Forward		GGTTTTGTGTCTGAATGTGGA	64
		Reverse		CCATGCACATTTCCCATAAT	
		Single base extension	40-Y	GTCTGAATGTGGATAATATATGG	
			41-Y	TTCCCATAATCAACATGAGGC	
5	SNP-PIS-136404	Forward		GAATTCTTTTAGGGGTTGTCA	67
		Reverse		CCAGCTTTAGAGTTGGCAAT	
		Single base extension	23-K	GAATTCTTTTAGGGGTTGTCAAA	
6	SNP-PIS-167992	Forward		CGAAAAATAACTTCATAGCGTGA	69
		Reverse		TGATCAATTGCAGCTTAGGG	
		Single base extension	36-W	AGCTTAGGGTTGCGGTTA	
7	SNP-PIS-174431	Forward		AGTCTCTAGCCGCGTACATA	64
		Reverse		AACTTAACAATTTYTCCACTTTCAC	
		Single base extension	56-M	CCGCGTACATATAAAAATTAACTACTCA	

According to the SNaPshot analysis of 13 loci, eight SNP loci could successfully separate sex in pistachio. There were two adjacent loci in two reads (SNP-PIS-133396 and SNP-PIS-135862) within the 13 candidate loci. Both SNP loci in SNP-PIS-133396 separated sexes, whereas one of the SNP loci in SNP-PIS-135862 was ineffective in sex determination (Table 4). Five of the loci were unable to distinguish F_1_ individuals and were discarded; further analysis continued using the remaining eight SNP loci from seven reads. Therefore, seven pairs of SNP flanking primers were used in the HRM analysis. Since, HRM analysis is another SNP genotyping method for rapid and cost-effective analysis of genetic variation within PCR amplicons. The SNaPshot analysis results for the eight tested sex-linked loci revealed that all female individuals were heterozygous and all male individuals were homozygous (Table 4).

**Table 4:** **Results of SNaPshot and HRM analysis in sex-linked SNP markers in pistachio**

**SNP loci**	**SNP position**	**SNaPshot**	**HRM**	**Genotype in female**	**Genotype in male**
SNP-PIS-112277	61R	+	-	R (TC)	T
SNP-PIS-127343	27S	+	-	S (GC)	G
SNP-PIS-133396	46S	+	+	S (GC)	G
	47Y	+		Y (GA)	G
SNP-PIS-135862	41Y	+	-	Y (GA)	A
SNP-PIS-136404	23 K	+	+	K (AC)	C
SNP-PIS-167992	36 W	+	+	W (TA)	A
SNP-PIS-174431	56 M	+	+	M (CA)	C

**Figure 1:**
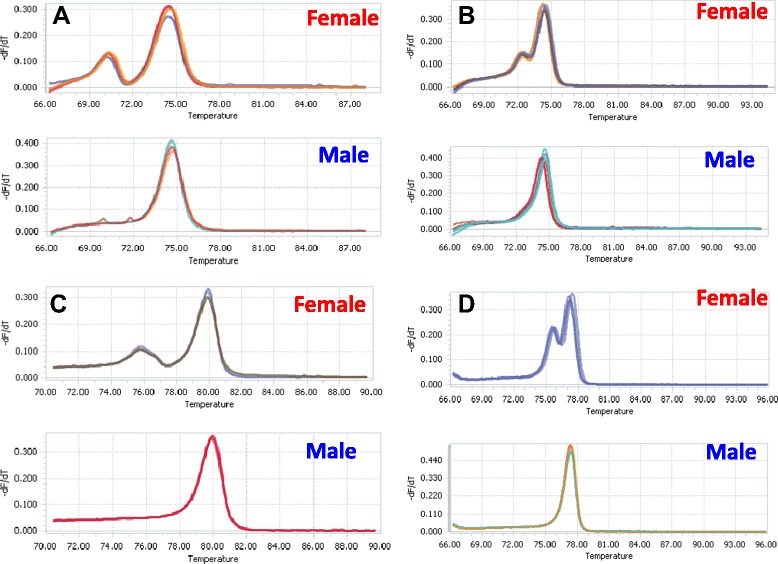
**Derivative melting curves of four SNP sex-linked loci in five female and in five male individuals. (A)** SNP-PIS-133396, **(B)** SNP-PIS-136404, **(C)** SNP-PIS-167992, and **(D)** SNP-PIS-174431. Y axis show negative derivative of normalized fluorescence with respect to temperature (−d*F*/d*T*) and X axis shows temperature. Female and male DNAs were amplified in Light Cycler 96 Real-Time PCR system using Syto 9 dye.

HRM analysis was performed in the same plants used for SNaPshot analysis. Among the seven tested SNP loci, four (SNP-PIS-133396, SNP-PIS-136404, SNP-PIS-167992, SNP-PIS-174431) could distinguish sex in all 166 tested plants (Table 4, Figure 1). The remaining three SNP loci were unable to distinguish sex by HRM analysis. Therefore, four loci were identified as being suitable for marker-assisted selection (MAS) in breeding programs. Again, all female individuals were heterozygous and all male individuals were homozygous in the HRM analysis results of the four sex-linked loci.

### Testing sex-linked SNP markers in wild *Pistacia* species

To test the eight SNP loci by SNaPshot analysis in four wild *Pistacia* species, 10 female and 10 male individuals were assessed from *P. atlantica* (Desf.)*, P. terebinthus* L., *P. eurycarpa* Yalt.*,* and *P. integerrima* Stewart, respectively. SNaPshot analysis revealed that none of the sex-linked markers in *P. vera* could separate sex in the tested wild *Pistacia* species.

## Discussion

### RADseq in pistachio

From 18 pistachio plants, 36.96 Gb of data was generated by restriction site-associated DNA sequencing (RADseq) on the Hi-Seq 2000 next-generation sequencing (NGS) platform, and the average data per pistachio plant was 2.05 Gb. Yang et al. [12,13] previously used the same methodology and sequencing platform to identify markers linked to anthracnose and stem blight in lupin. The authors generated 17.33 Gb data from 20 plants, with an average of 0.87 Gb data per plant. Therefore, this study made use of more than double the quantity of data generated for marker discovery than was used for lupin.

The number of RAD tags in this study was 9.36 million, with an average of 519,937 per plant, whereas Yang et al. [13] generated 7.6 million RAD tags at an average of 381,527 per plant. Average coverage of the RAD tags was 47.3×, demonstrating the reliability of the generated data in this study. According to flow cytometry analysis, the haploid genome size of pistachio is ~660 Mbp. [8]. Therefore, it is easy to calculate the sequence coverage of each plant used in this study, with an average of 3.1× coverage per plant obtained. The high-sequencing coverage depth of plants demonstrates the high reliability of generated SNP markers in this study; higher sequencing coverage is required for the discovery of reliable markers in heterozygous populations than in homozygous ones.

### Identification of sex-linked SNP markers in pistachio

This study identified eight SNP sex-linked loci for pistachio from seven reads using RADseq at three stages: (1) putative sex-linked SNP loci discovery, (2) validation of sex-linked SNP markers in a large germplasm by SNaPshot analysis, and (3) cost-effective application of sex-linked SNP markers by high-resolution melting (HRM) analysis.

In the first stage, 38 putative sex-linked SNP markers were identified among the 33,757 polymorphic SNP markers produced from 36.96 Gb of data by RADseq on the Hi-Seq 2000 NGS platform. Using the same platform and methodology, Yang et al. [12] obtained 8,207 polymorphic SNP markers in lupin for the development of markers linked to anthracnose, and the authors identified 38 candidate-linked markers. In the second RADseq study by Yang et al. [13] to identify markers linked to stem blight in lupin, 7,241 polymorphic markers were generated, and 33 candidate-linked markers found. The number of generated candidate markers was similar in pistachio and lupin for different traits. However, there were 4.5 times more polymorphic SNP loci in pistachio than in lupin, this is probably owing to the high heterozygosity level of plant materials used for our study; we used an F_1_ population in a dioecious species whereas the lupin studies used a RIL population.

In the second stage, validation of putative sex-linked markers was performed by SNaPshot analysis in three different plant materials: (1) 42 segregating mature F_1_ progenies (26 were male, 16 were female) from a Siirt × Bağyolu population, (2) 17 male and 47 female pistachio cultivars or genotypes in the germplasm collection, and (3) 60 open-pollinated mature pistachio trees (30 were male, 30 were female) derived from Siirt and Ohadi cultivars. Among 38 putative sex-linked SNP loci from 28 RAD reads, 13 allowed for primer design and eight distinguished pistachio sex with complete accuracy when using SNaPshot analysis.

In the SNaPshot analysis, one of the eight sex-linked SNP loci reads had adjacent SNP loci. Therefore, only SNP flanking primers from seven reads were used for the third stage, in which HRM analysis was performed to demonstrate cost-effective use of sex-linked markers in a pistachio breeding program. Among these seven, four SNP flanking primer pairs were able to separate the sexes in pistachio, and are considered potential sex-linked markers for MAS in pistachio breeding applications.

In an earlier study, performed by Hormaza [33] in *P. vera*, a single female-specific sex marker (OPO08_945_) was found by screening 1,000 Ramdomly Amplified Polymorphic DNA (RAPD) primers. Yakubov *et al.* [34] converted the OPO08_945_ RAPD marker into a Sequence Characterized Amplified Region (SCAR) marker in combination with touchdown PCR for reliable screening; however, testing of the SCAR primer in our laboratory in the segregating population, and in a large germplasm collection, generated false negatives in some female individuals and false positives in some male individuals, including the Bağyolu male parent. The reason for the discrepancy between this study and earlier ones may be the testing of markers in a limited number of individuals. For example, Hormaza *et al.* [32] tested the marker in seven male and seven female cultivars. It is therefore necessary to test candidate markers in a large germplasm collection for validation of reliable markers. Consequently, new, fully reliable, sex-linked markers were generated in this study for use in pistachio.

### Heterogametic female sex-linked markers suggest ZW/ZZ sex determination in pistachio

In some plant species, sex determination is based on the presence of either homomorphic, (e.g., in *Carica papaya*), or heteromorphic sex chromosomes (e.g., in *Silene latifolia*). Recombination in sex determination regions in these plants is suppressed [25,36]. The suppressed recombination leads to accumulation of repetitive sequences, inversions, deletions, or translocations in different plant species [25,37,38].

Identification of 38 putative sex-associated SNP markers as heterozygous in female individuals and homozygous in male individuals using RADseq suggests a ZW/ZZ sex determination system in pistachio. Validation of the markers by SNaPshot analysis, and the female-specific marker identified by Hormaza *et al.* [32] support this hypothesis. Cherif *et al.* [39] found three genetically linked simple sequence repeat (SSR) markers that are heterozygous only in male individuals, and suggested an XY chromosomal system with a non-recombining XY-like region in date palm (*Phoenix dactylifera*). Carmichael *et al.* [21] used RADseq technology to find sex-linked markers in the salmon louse (*L. salmonis*). They identified one SNP marker that was heterozygous in female individuals and homozygous in male individuals, and suggested a ZW/ZZ female heterogametic sex determination system. Palaiokostas *et al.* [20] used RADseq for mapping and sex determination in *H. hippoglossus*, and revealed XX/XY sex determination. In this study, we identified eight sex-linked loci that were all female heterogametic, further suggesting a ZZ/ZW sex determination system in pistachio.

**Figure 2:**
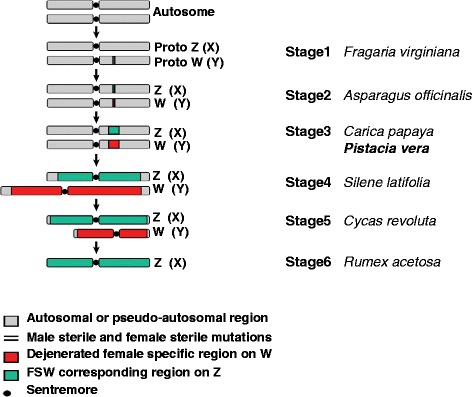
**The six stages of sex chromosome evolution obtained from Ming et al. (30) and adapted to ZW system.** Stage 1: Unisexual mutation with complementary dominance. Stage 2: Recombination is suppressed between the two sex determination loci and WW genotype is viable. Stage 3: Suppression of recombination extends to neighboring regions and form a small female-specific region of the W chromosome (FSW). Stage 4: The FSW region expands in size, and the Z and W chromosomes become heteromorphic. Stage 5: Severe dejeneration of the W chromosome causes deletion of nonfunctional DNA sequences, and results in reduction of W-chromosome size. Stage 6: Suppression of recombination spreads to the entire W chromosome, W chromosome is totally lost, and Z-to-autosome ratio sex determination system has evolved.

A new scenario has been widely accepted for describing the evolution of sex chromosomes (Figure 2). This comprises six consecutive steps, and was developed following extensive genetic and genomic studies on the male-specific region [30,40-42]. *Actinidia chinensis* is in the second stage, and *Carica papaya* is in the third stage. Pistachio may be in stage 2 or 3, depending on whether the WW female genotype is viable. The reported female-to-male ratio in the segregated populations is 1:1, and there is no report of super-female genotypes in pistachio, this suggests that the WW genotype is not viable. We therefore propose the evolution of sex chromosomes at stage 3, where suppression of recombination extends to neighboring regions allowing a large number of W-linked genes to degenerate and form a female-specific region on the nascent W chromosome, such as the one observed in *C. papaya* [30].

In papaya, 225 of the 342 markers on linkage group 1 co-segregate with the sex locus, indicating severe suppression of recombination at this region [43]. Similar results were obtained in *Asparagus* [25], *Humulus* [44], *Silene* [45], and *Spinacia* [46]. We also had similar results, having eight sex-linked loci from 13 reads, indicating that suppression of recombination occurred in pistachio. RADseq SNP markers are derived from a reduced genome and the sex-linked SNP loci indicate the formation of a non-recombining sex-determining region in pistachio. Suppression of recombination in specific chromosomal regions is a widespread phenomenon in sexually reproducing organisms, and has been documented in plant species with both primitive and advanced sex chromosomes [30,43,44,47].

### Testing sex-linked SNP markers in wild *Pistacia* species

SNaPshot analysis of eight SNP loci in four wild *Pistacia* species revealed that sex-linked markers in *P. vera* could not distinguish sex in the four tested species. Hormaza [33] also tested a female-specific RAPD marker in wild *Pistacia* species and found the marker ineffective for sex determination. However, Esfandiyari *et al.* [35] recently tested the marker in four *P. atlantica* and four *P. khinjuk* individuals and reported that the SCAR marker converted by Yakubov *et al.* [34] effectively distinguishes sex in wild species. It is noteworthy that testing of RAPD and SCAR primers developed by Hormaza *et al.* [32] and Yakubov *et al.* [34] in four wild *Pistacia* species in our study was unable to distinguish sex in wild *Pistacia* species. Therefore, validation of markers in a large germplasm is one of the most important aspects of marker development.

It is interesting that none of the sex-linked markers identified in *P. vera* by Hormaza [33] or in this study are unable to separate the sexes in wild species. It will be interesting to identify markers for the wild species and to create a sex determination system for them. Mapping of sex-linked SNP markers in an intra- and inter-specific F_1_ segregating population, which may show the positions of markers in the maps of cultivated and wild *Pistacia* species, is underway.

## Conclusions

Restriction site-associated DNA sequencing (RADseq) technology was used to identify sex-linked markers in pistachio. Thirty-eight putative sex-linked markers were detected from 28 reads following RADseq. Thirteen single nucleotide polymorphism (SNP) loci were suitable for primer design, and eight of these could distinguish sex with complete accuracy in pistachio following validation in mature F_1_ progenies, and in a large germplasm collection by SNaPshot analysis. Sex-linked SNP markers were tested by high-resolution melting (HRM) analysis with real-time PCR for cost-effective application in a cultivar-breeding program, and four of them perfectly distinguished sex. Female heterogamety of all sex-linked SNP markers suggests a ZZ/ZW sex determination system in pistachio. The reported female-to-male segregation ratio of 1:1 in all reported segregated populations, and no reports of super-female genotypes or heteromorphic sex chromosomes in pistachio suggests that the WW genotype is not viable. We therefore propose that the evolution of the sex chromosome is at stage 3, where a pair of primitive sex chromosomes controls sex determination, with a female-specific region on the W chromosome.

## Methods

### Plant material and DNA extraction

An F_1_ population between female Siirt and male Bağyolu cultivars was used for marker development. The population consisted of 91 F1 progenies planted in 2004 in Gaziantep province, Turkey. Forty-two flowered by the spring of 2014, and rest of the progenies remained in the juvenile stage. DNA extractions were based on the CTAB protocol [48], with minor modifications [4]. The quantification of DNA samples was performed using a Qubit fluorometer (Invitrogen, Carlsbad-CA).

### Identifying markers linked to sex by RADseq in pistachio

Eighteen plants were used for restriction site-associated DNA sequencing (RADseq) for sex-linked marker development in pistachio: eight F_1_ female individuals, eight F_1_ male individuals, and the two parents from a Siirt × Bağyolu F_1_ segregating population. The RADseq protocol was similar to that previously described in the literature [9,11,12]. *EcoRI* (recognition site 5′-G/AATTC-3′) was used as a restriction enzyme in the protocol. Then, 90-bp pair-end (PE) sequencing libraries were constructed using eight-nucleotide multiplex identifiers (MIDs). Each plant was treated separately, and was assigned a unique MID barcode. Each sex group of individuals was sequenced in different lanes of a Hi-Seq 2000 sequencing platform (Illumina). Library construction and sequencing were performed at the Beijing Genomic Institute, China. The length of the first reads, including the MID barcodes, was 90 bp. Following RAD, reads were assigned to individual plants, and the eight-nucleotide MID barcode sequences removed. The length of the first RAD reads (RAD loci) was 82 bp, this did not include the first nucleotide G of the *EcoR*1 recognition site. The 82-bp first reads within each individual plant were grouped into tag reads based on sequence similarity by allowing a maximum of two mismatches between any two RAD reads. Monomorphic DNA sequences were removed by comparison and filtering of tag reads from the parents. Only first reads containing polymorphic SNP between the two parents were kept. The remaining sequences were compared among all the plants to identify sex-specific SNP loci. If a SNP marker showed a polymorphic locus correlating with the sex of the individuals, it was regarded as a sex-associated marker in pistachio.

### Primer design

SNP flanking and single-base extension primers were designed using BatchPrimer 3 web based software [49] for SNaPshot and high-resolution melting (HRM) analysis (Table 3). Primer design was performed in the first RAD reads if DNA sequences were suitable. Single-base extension primers were designed adjacent to the SNP loci from one of the orientations (forward or reverse) if the sequences were appropriate. Standard parameters of BatchPrimer 3 web based software were used for primer design.

### Validation of sex-associated SNP markers by SNaPshot analysis

Testing of sex-associated SNP markers was initially performed in all 42 mature F_1_ plants in the Siirt × Bağyolu population. Then, if they succeeded to distinguish the sex of F_1_ progenies, they were tested using 17 male and 47 female cultivars in the germplasm collection previously characterized by Kafkas *et al.* [50] to validate marker-assisted selection (MAS) markers for a pistachio cultivar-breeding program. Additionally, markers were tested in 30 male and 30 female open-pollinated mature progenies of Siirt and Ohadi cultivars.

Before the SNaPshot analysis, PCR amplifications were performed with SNP flanking primers in a reaction volume of 25 μL containing 75 mM Tris–HCl (pH 8.8), 20 mM (NH_4_)_2_SO_4_, 2 mM MgCl_2_, 0.1% Tween 20, 100 μM each of dATP, dTTP, dGTP, and dCTP, 0.2 μM each of reverse and forward primers, 1.0 unit Hotstart *Taq* DNA polymerase (Thermo Scientific, Vilnius-Lithuania), and 20 ng of genomic DNA. Thermal cycler (Veriti, Applied Biosystems Inc, Singapure) conditions were: 2 min at 94°C for pre-denaturation; 35 cycles of 45 s at 94°C for denaturation, 45 s at 50°C for annealing, and 1 min at 72°C for extension; followed by a final incubation of 5 min at 72°C. Next, 5 μL of PCR product was treated with 1 unit of shrimp alkaline phosphatase and 10 units of exonuclease I (Thermo Scientific, Vilnius-Lithuania) at 37°C for 30 min to remove excess dNTPs and primers, respectively. The product was then incubated for 10 min at 85°C for inhibition and inactivation.

SNaPshot analysis was performed using an Applied Biosystems SNaPshot Multiplex Kit [Applied Biosystems (ABI) Inc., UK] according to manufacturer protocol with minor modifications. Reactions were performed in a volume of 10 μL, containing 5 μL SNaPshot Ready Multiplex Ready Reaction Mix, 0.5 μM single-base extension primer, and 1 μL shrimp alkaline phosphatase/exonuclease-treated PCR product. The thermal cycler conditions were 10 s at 96°C; 30 cycles of 10s at 96°C, 5 s at 50°C, and 30s at 60°C; followed by 2 min at 60°C. Labeled extension products were treated with 1 unit shrimp alkaline phosphatase. One microliter of diluted extension product was mixed with 9.8 μL Hi-Di formamide and 0.2 μL GeneScan-120 LIZ Size Standard [Applied Biosystems (ABI) Inc., Foster City, CA]. Products were denatured at 95°C for 5 min and electrophoresis performed using an ABI PRISM 3130xl Genetic Analyzer [Applied Biosystems (ABI) Inc., Tokyo-Japan] with a 36-cm-length capillary array and POP-7 polymer [Applied Biosystems (ABI) Inc., Foster City, CA]. Data analysis was performed using GeneMapper 4.0 software (ABI).

### HRM analysis

If markers passed the validation process described above for use in MAS in breeding programs, then HRM analysis was performed using a Light Cycler 96 Real-Time PCR instrument (Rosch, Mannheim-Germany) with two replicates. The HRM amplification reactions were carried out in a total volume of 20 μL containing 15 ng DNA, 75 mM Tris–HCl (pH 8.3), 20 mM (NH_4_)_2_SO_4_, 2.5 mM MgCl_2_, 0.1% Tween 20, 200 μM each of dATP, dTTP, dGTP, and dCTP, 0.25 μM each of reverse and forward primers, 1.0 unit Hotstart Taq DNA polymerase, and 1.5 μM Syto 9 dye (Life Technologies, Carlsbad-CA). The cycling program was: pre-denaturalization for 600 s at 95°C; 45 cycles of 95°C denaturalization for 10 s, 60°C annealing for 15 s, and 72°C extension for 15 s. Amplification cycles were immediately followed by HRM steps of 95°C for 60 s, cooling to 40°C for 60 s, then raising the temperature to 65°C and then 97°C for 15 s. The annealing temperature was decreased in subsequent cycles by 0.5°C per cycle after the first 60°C annealing step, down to 55°C.

### Testing sex-linked markers in wild *Pistacia* species

SNP markers that distinguished sex in *P. vera* with 100% accuracy were screened in 20 individuals (10 males and 10 females) of the following wild *Pistacia* species: *P. atlantica, P. eurycarpa, P. terebinthus,* and *P. integerrima* using SNaPshot analysis.

## Abbreviations

RADseq: Restriction site-associated DNA sequencing
SNP: Single nucleotide polymorphism
MAS: Marker-assisted selection
SCAR: Sequenced Characterized Amplified Region
HRM: High-resolution melting
